# A large primary leiomyoma of right ventricle in a 4-year-old child: case report

**DOI:** 10.3389/fcvm.2025.1578776

**Published:** 2025-08-20

**Authors:** Yefu Liu, Dou Yuan

**Affiliations:** ^1^ Department of Cardiology, Chengdu Shang Jin Nan Fu Hospital, West China Hospital of Sichuan University, Chengdu, Sichuan, China; ^2^ Department of Cardiovascular Surgery, Chengdu Shang Jin Nan Fu Hospital, West China Hospital of Sichuan University, Chengdu, Sichuan, China

**Keywords:** cardiac tumor, primary cardiac tumor, leiomyoma, right ventricle, child

## Abstract

**Background:**

Cardiac leiomyomas are extremely rare cardiac anomalies. Among them, primary leiomyomas confined to heart are even rarer, which have been reported only in few cases. Once diagnosed, surgical resection is the most common choice for treatment.

**Case summary:**

A 4-year-old girl with heart murmur was diagnosed with a large spherical mass which was located in the right ventricle. The oval mass measured approximately 50 × 40 mm and caused obstruction of the right ventricular outflow tract. To relieve the obstruction, the large mass was resected in surgery. During the surgery a 45 × 40 × 35 mm oval structure with stiff elastic consistence was found being attached to the free wall of the right ventricle. No other abnormalities like regurgitation were detected. Histopathological examination of the resected mass indicated a benign leiomyoma. Postoperative recovery was uneventful.

**Discussion:**

As a benign cardiac tumor, leiomyoma is exceptionally rare. The majority of cardiac leiomyomas are metastatic. Primary ones are few. Imaging and histologic examination can help to diagnose. After surgical resection, most patients can recover well.

## Introduction

Cardiac tumors are an uncommon type of heart disease, especially primary cardiac tumors (1, 2). Among cardiac tumors, most cardiac leiomyomas are metastatic, with only a few cases of primary cardiac leiomyomas reported in the literature (3, 4). Commonly, cardiac leiomyomas can lead to heart murmur, obstruction or regurgitation, because of its occupation of cardiac cavity and pression to intracardiac structure (5–7). As for treatment, surgical resection is an effective method to achieve good prognosis. Herein, we report a case of a large primary leiomyoma in right ventricle in a child, which is extremely rare. To relieve the obstruction of the right ventricular outflow tract, tumor resection was performed and the patient recovered well. We write to highlight this case and share our experience of treating such a rare cardiac tumor.

## Case presentation

**Figure 1:**
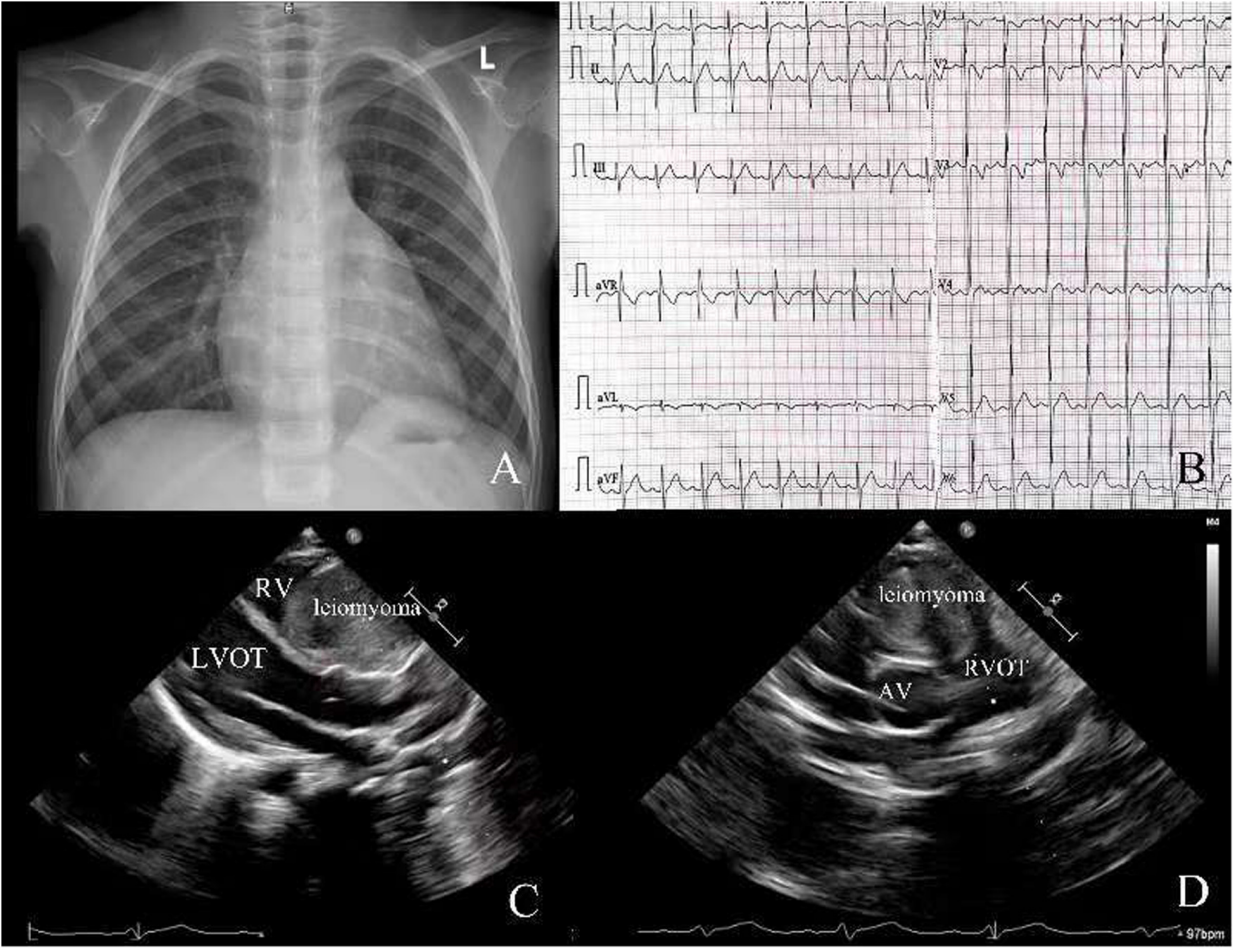
**(A)** Chest x-ray before surgery; **(B)** electrocardiogram before surgery; **(C,D)** transthoracic echocardiography before surgery. RV, right ventricle; LVOT, left ventricular outflow tract; RVOT, right ventricular outflow tract; AV, aortic valve.

**Figure 2:**
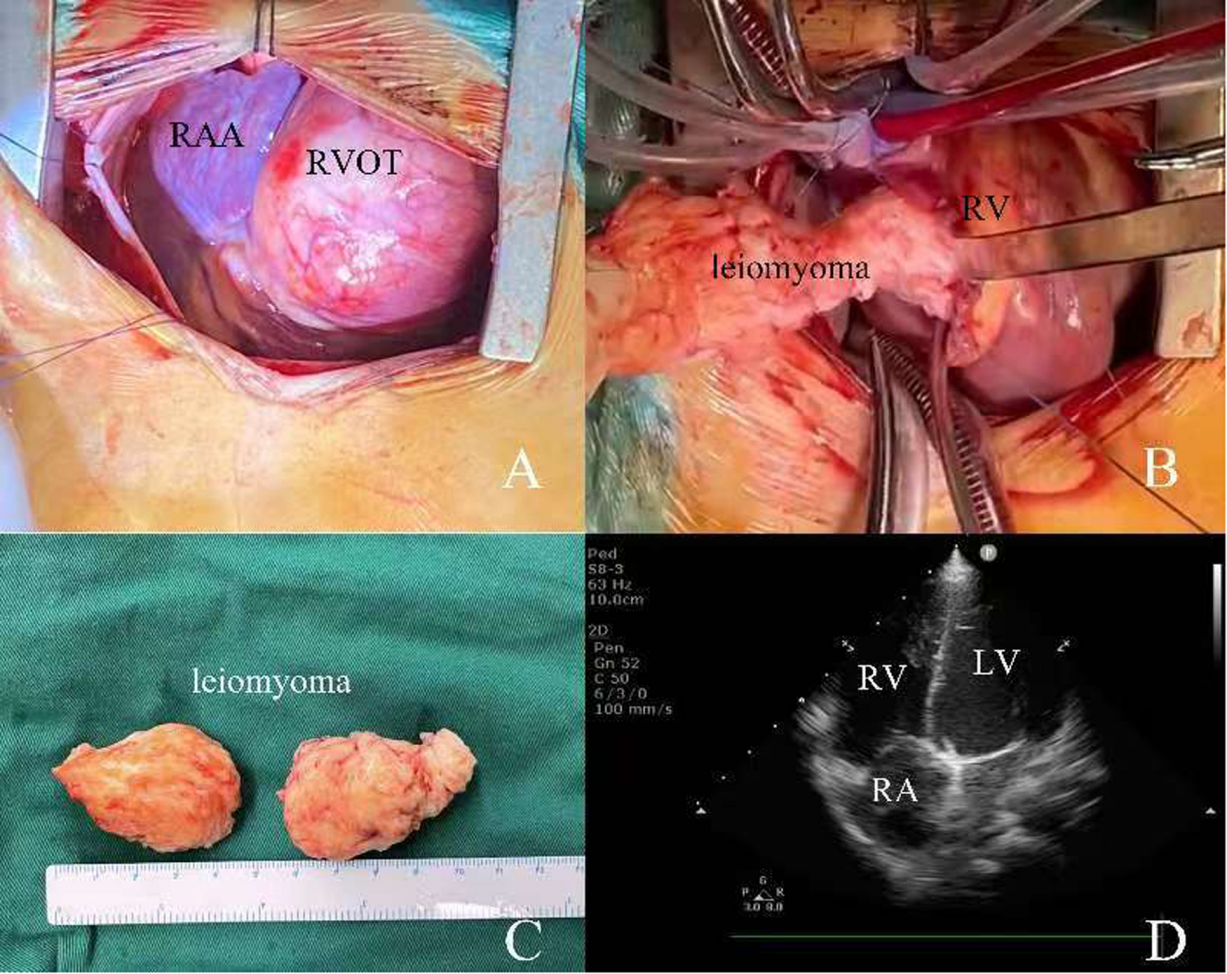
**(A,B)** intraoperative view of RVOT and the resection of leiomyoma; **(C)** the size of resected leiomyoma; **(D)** transthoracic echocardiography after surgery. RAA, right atrial appendage; RA, right atrium; RV, right ventricle; RVOT, right ventricular outflow tract.

A 4-year-old girl was admitted to our hospital due to a heart murmur in the second and third left intercostal space along the left sternal border, which was incidentally revealed by physical examination during her treatment for a cold. According to the patient and her parents, she had no other symptoms except for a grade III/VI systolic murmur best heard at the left upper sternal border during mid-systole, consistent with turbulent flow across the right ventricular outflow tract**.** The electrocardiogram showed sinus rhythm (Figure 1B). The chest x-ray also demonstrated a normal heart shadow (Figure 1A). Transthoracic echocardiography revealed a large spherical mass measuring approximately 50 × 40 mm which was located in the right ventricle leading to outflow tract obstruction (Figures 1C,D). The tricuspid valve and pulmonary valve functioned well, and no obvious regurgitation was detected. The pre-operative echocardiogram showed marked acceleration in the right ventricular outflow tract, with a peak velocity of 4 m/s and a significant elevation in right ventricular pressure, which is why surgical treatment was undertaken. Because the tumor was initially presumed to be benign and the patient was too young to cooperate with an MRI, we did not perform this examination. After adequate preoperative preparation, the patient underwent surgery to resect the large mass and relieve the obstruction of the right ventricular outflow tract. The operation was performed under cardiopulmonary bypass. During the surgery, the right ventricular outflow tract presented as an obvious spherical expansion (Figure 2A). Through the incision upon the surface of right ventricular free wall, the inner structure of right ventricle, tricuspid valve and pulmonary valve were checked. In the right ventricle, a mildly movable oval mass with a stiff elastic consistence, measuring 45 × 40 × 35 mm, was attached to the free wall of the right ventricle. With intact capsule, it occupied a large amount of cavity of right ventricle and obstructed the right ventricular outflow tract. The tumor was surgically removed in its entirety and completely detached without any appreciable intra-operative hemorrhage. After tumor resection, 2 cm × 2 cm autologous pericardial patch was used to reconstruct the right ventricular outflow tract. The structure of tricuspid valve and pulmonary valve was normal. Along the border between normal ventricular wall and the mass, it was resected (Figures 2B,C). Water injection test indicated normal valve function, and the incision was conventionally closed. Histopathological examination of the resected tissue from the mass demonstrated that it was composed of spindle-shaped cells and interstitial collagen similar to which in benign leiomyoma. Atypia and necrosis were absent. Postoperative transthoracic echocardiography showed that the cavity of right ventricle was clear (Figure 2D). On the sixth day after surgery, having completed all re-examinations, the patient was discharged. Postoperative recovery was uneventful. At 6 months, 1 year, and 3 years postoperatively, all follow-up visits included echocardiography and ECG. The ECG remained normal on every occasion. Echocardiography showed a patent right ventricular outflow tract with a flow velocity of 0.8 m/s and no regurgitation of the pulmonary or tricuspid valves.

## Discussion

The present case sharpens the clinical lens on two seldom-encountered entities: primary cardiac leiomyoma and pediatric right-sided intracavitary tumors. Whereas the literature is dominated by intravenous leiomyomatosis of uterine origin that creeps cephalad into the right heart, this 4-year-old child had no extra-cardiac disease, no hormonal driver, and a tumor that was sessile rather than worm-like. These features redirect attention from metastatic pathways to de-novo transformation of intramyocardial smooth-muscle cells, a mechanism supported by the absence of estrogen/progesterone receptors on immunohistochemistry. Leiomyoma is a benign tumor of mesenchymal origin. The majority of leiomyomas are involved in uterus. In some rare cases, the uterine leiomyoma might have distant metastasis (9–11). Metastases of leiomyomas have been found in veins, lungs, paraaortic lymph nodes, abdominal lymph nodes, heart, breasts, and so on (12, 13). Most cardiac leiomyomas are metastatic (5, 14). They occur predominantly in middle-aged women with a mean age of 47 years old (4). These leiomyomas often originate in the uterus, transfer into inferior vena cava, and form a continuous mass into the right atriums and ventricles (15–19). They can also directly transfer into right ventricle and form intracardiac leiomyomas (20–24). Most of these tumors are mobile and hardly adhere to any wall of the ventricle (25).

Diagnostic reasoning in a child with a new murmur normally privileges rhabdomyoma or fibroma; however, the rapid rise in RVOT gradient (4 m/s) and the spherical, encapsulated mass on echocardiography mandated early surgical extirpation. Delay would have risked acute obstruction or irreversible RV remodeling. MRI was forgone because of age and the urgency of symptoms, underscoring that echocardiography alone can be decisive when the index of suspicion is adjusted for rarity (6, 26–29).

Technically, the broad-based attachment required en-bloc resection with a 2 mm myocardial cuff to ensure true oncologic margins, followed by autologous pericardial patch reconstruction to accommodate future somatic growth and avoid prosthetic mismatch. The immediate fall in RVOT gradient from 64 mmHg to 3 mmHg confirms that complete excision is both feasible and curative in this setting. Most patients with intracardiac leiomyomas have different symptoms, most common of which are heart murmur, valve regurgitation and obstruction. Like in this case, the leiomyoma is often large and can occupy the space of the cardiac chamber, leading to obstruction and heart murmur (1, 7). When the mass protruded into another chamber or pulmonary artery trunk, it will cause valve insufficiency (7, 26). Besides the size of leiomyomas, different locations of the leiomyomas may also lead to different symptoms.

In terms of diagnosis, echocardiography, computed tomography, and MRI are highly effective in detecting tumors (8, 25). Furthermore, to determine the nature of tumors, a histologic examination is necessary. In the process of histologic examination, cell morphology, atypia, necrosis, smooth muscle actin, progesterone and estrogen receptors are important indicators (6, 23, 24).

Long-term surveillance is minimal but not trivial: annual echocardiography for 5 years suffices to exclude the vanishingly small risk of local recurrence or patch aneurysm. No adjuvant therapy is indicated, and the child remains asymptomatic—evidence that prompt recognition and radical yet conservative surgery convert an otherwise obscure lesion into a benign footnote in an otherwise healthy life (30).

## Conclusion

Primary right ventricular leiomyoma is an extremely rare disease. Imaging examinations and histological examinations can help to make a definite diagnosis. When treated properly, patients with primary right ventricular leiomyoma can achieve a good prognosis.

## Data Availability

The raw data supporting the conclusions of this article will be made available by the authors, without undue reservation.
